# A longitudinal roadside study of the New Hampshire alder root nodule microbiome

**DOI:** 10.1128/aem.00446-26

**Published:** 2026-05-27

**Authors:** Alexandra Gomez, Louis S. Tisa

**Affiliations:** 1 Department of Molecular, Cellular and Biomedical Sciences, University of New Hampshire 3067, Durham, New Hampshire, USA; The University of Tennessee Knoxville, Knoxville, Tennessee, USA

**Keywords:** actinorhizal symbiosis, microbiome, root endophytes, seasonality, roadside, soils

## Abstract

Actinorhizal plants are pioneer plants that colonize harsh environments and have been used for land reclamation. Their ability to thrive under these harsh conditions is due to their symbiotic association with the nitrogen-fixing bacterium *Frankia* and forming a root nodule structure. Although the plant root nodule primarily contains the symbiont *Frankia*, other members of the nodule community have been identified. This study represents an investigation of the effects of different environments on the nodule microbiome of *Alnus rugosa,* a shrub actinorhizal tree found at different locations within New Hampshire over a 3-year period. We utilized 16S rRNA and ITS amplicon sequencing to map the seasonal bacterial and fungal communities in the *A. rugosa* root nodule microbiome compared to rhizosphere and bulk soil communities. The relative abundance of *Frankiaceae* in root nodules fluctuated seasonally and by site. Sites with lower relative abundance of *Frankiaceae* in nodules had higher relative abundances of fungal nodule occupants. The roadside bacterial communities were distinct from those at the rural site, with *Chitinophagaceae* and *Nitrosomonadaceae* being characteristic members of the roadside rhizospheres and bulk soils, respectively. Soil zinc significantly affected all microbial communities. Our results indicate that the *A. rugosa* root nodule and soil microbiomes are responsive to different environmental variables like roadways and other microorganisms, and these responses need to be further elucidated for the optimization of future *in situ* actinorhizal projects.

**IMPORTANCE:**

Actinorhizal plants like alders are important ecologically and economically as pioneering plants. The symbiotic association with Frankia greatly accelerates the growth of the host plant and indirectly does the same for neighboring plants. Actinorhizal trees provide an excellent mechanism to restore disrupted environmental sites and have been used to reclaim land that has been used for strip-mines, gravel pits, and soil stabilization of other landscapes disturbed by the effects of erosion and water runoff. Actinorhizal plants are found on coastal lands around the estuaries, and some are proven to be salt tolerant. Elucidating the dynamics of microbial community structure of the alder root nodules will help our understanding of the ability of these pioneering plants to reclaim degraded lands and to survive in diverse harsh environments. The role that other members of actinorhizal plant root nodule plays may be important to that survival ability. This field study reports on the influence of soil variables, habitats, and seasons on the dynamics of the actinorhizal microbiome.

## INTRODUCTION

Alder trees are a widely distributed genus of trees often found in riparian habitats. As a member of actinorhizal plants, these pioneer plants colonize a variety of different ecosystems including those with poor and polluted soils as well as areas burned by wildfires (1–3). This durability is due to their symbiotic association with the nitrogen-fixing soil bacterium *Frankia*. Alders also will form associations with ectomycorrhizae (ECM) and arbuscular mycorrhizae fungi, sometimes even simultaneously, for further aid in phosphorus and water acquisition (4, 5). Besides these symbionts, additional microbes may inhabit plant root nodules.

Actinorhizal nodules host communities of microbes, many of which play neutral to beneficial roles in the nodule environment. For example, two *Nocardia* species isolated from *Casuarina* nodules improved nodulation and plant growth rate in co-inoculation trials with *Frankia* (6). Mining of metagenomes from wild *Casuarina* nodules revealed potential genes for plant growth promotion from *Bacillus* and *Mycobacterium* genomes and siderophores from *Paenibacillus* (7). Another frequent inhabitant of actinorhizal nodules, *Micromonospora,* harbors plant growth hormones like indole acetic acid and phenylacetic acid, hydrolytic enzymes, and antagonistic properties toward several plant pathogens (7–9). There are also numerous reports of fungal endophytes like *Tomentella*, *Lactarius, Naucoria,* and *Cortinarius* though functional characterization of these fungi in wild actinorhizal nodules is needed (10, 11).

Root endophytic communities are dynamic. The growth cycles of host plants both influence and are influenced by their rhizosphere and root bacterial communities (12–15). Community profiling of soybean root nodules revealed shifts in the nodule communities under different watering regimes (16). *Frankia* abundance in actinorhizal host root nodules is also affected by low water availability (17). An exploration of the fluctuations in the nodule microbiome over time *in situ* is imperative: if future actinorhizal applications are to succeed, understanding what environmental factors may directly or indirectly alter the microbes within the system is necessary.

To understand the seasonal dynamics of the *Alnus rugosa* nodule microbiome and the impact of environmental conditions on these microbial communities, a 3-year study was performed on *A. rugosa* trees at six different sites in New Hampshire. The Candia site was the rural site as it was not near any roadways. The Candia site represents a pristine freshwater wetland site, while the Hampton site was adjacent to a saltwater marsh. The Raymond site was near a highway without a permanent water source, and the Epping site was an urban location near a highway. The Durham site was between a highway and freshwater wetland, and the Durham-adjacent site was more wooded. The sites differed in their geographical characteristics, with all but the Candia site being located next to major roadways, so we hypothesized that the roadway sites would have significantly different alder nodule and soil communities compared to the rural site due to the increase of heavy metals (18) and hydrocarbon contaminants from vehicular exhaust and runoff (19, 20). We cataloged the seasonal alterations in both the bacterial and fungal communities through 16S rRNA gene and internal transcribed spacer (ITS) amplicon sequencing, following the same wild trees for the entirety of the study.

## RESULTS

### Diversities of the alder bacterial and fungal microbiomes

**Fig 1:**
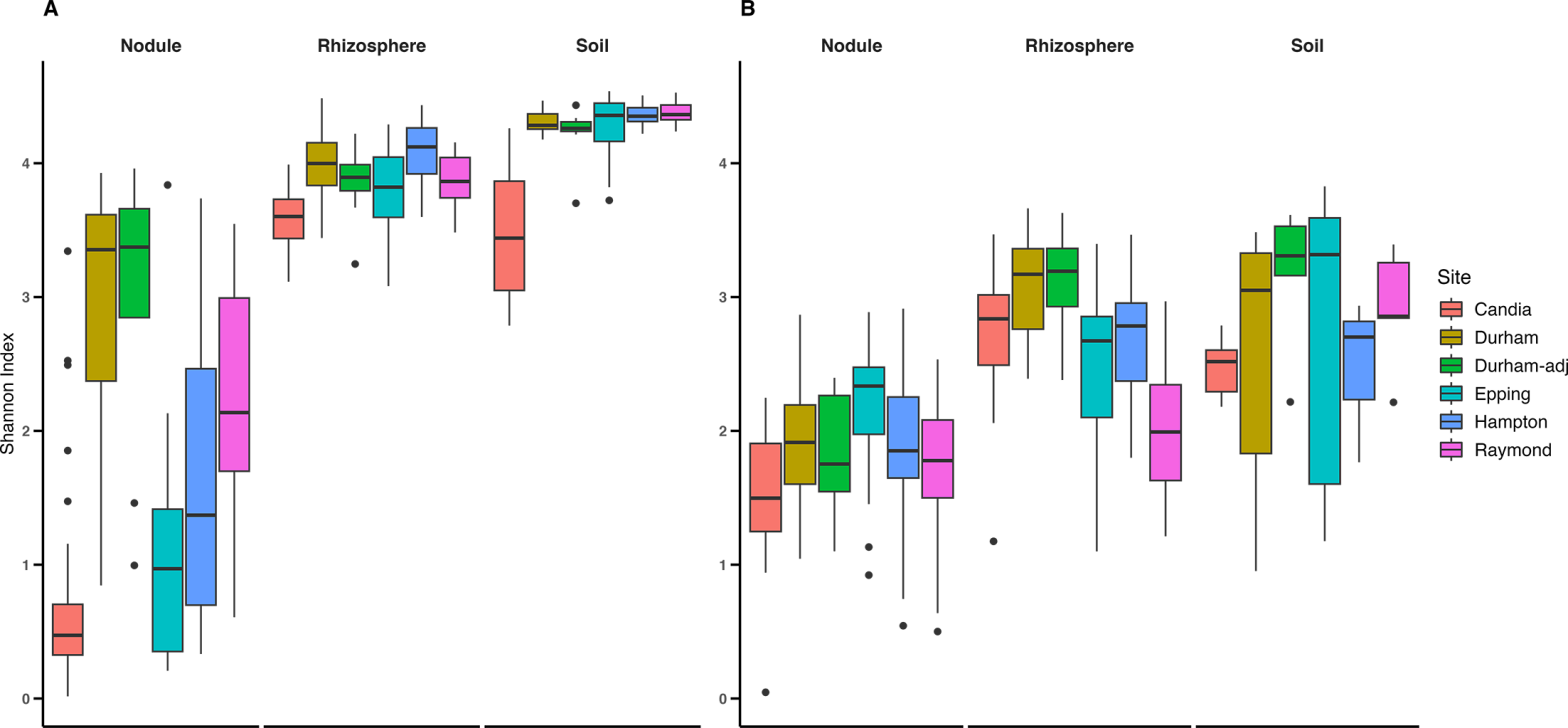
Shannon diversity index of the nodule, rhizosphere, and bulk soil bacterial (**A**) and fungal (**B**) communities across all sampling periods. Colors denote the different sampling sites.

At the rural Candia site, nodule, rhizosphere, and soil bacterial communities all had significantly lower Shannon diversity indices compared to the roadway sites, with the only exception being the Epping nodules (Fig. 1A) (Table S1). Shannon diversity indices were higher in the bacterial rhizosphere communities and were highest in the soil communities (Fig. 1A). Due to the loss of two Epping trees in October 2023 and the Hampton trees in April 2024, an additional Kruskal-Wallis test was conducted for the nodule communities at these sites. There was no statistical evidence that the Shannon diversity indices differed in the bacterial nodule communities between the original and new trees at the Hampton or Epping sites (*P* = 0.32 and *P* = 0.78, respectively). The yearly Shannon diversity indices for the bacterial nodule, rhizosphere, and soil communities at each sampling site can be seen in Fig. S1 through S3, respectively.

In the nodule fungal communities, less variation in Shannon diversity indices was seen between the rural Candia site and the roadway sites (Fig. 1B) (Table S2). The Raymond site’s rhizosphere fungal communities had significantly lower Shannon diversity indices compared to all other sites (Fig. 1B) (Table S2). There were no significant differences in the fungal nodule communities’ Shannon diversity indices between the original and new trees at the Hampton and Epping sites (*P* = 0.61 and *P* = 0.42, respectively). The yearly Shannon diversity indices for the fungal nodule, rhizosphere, and soil communities at each sampling site can be seen in Fig. S1, respectively.

**Fig 2:**
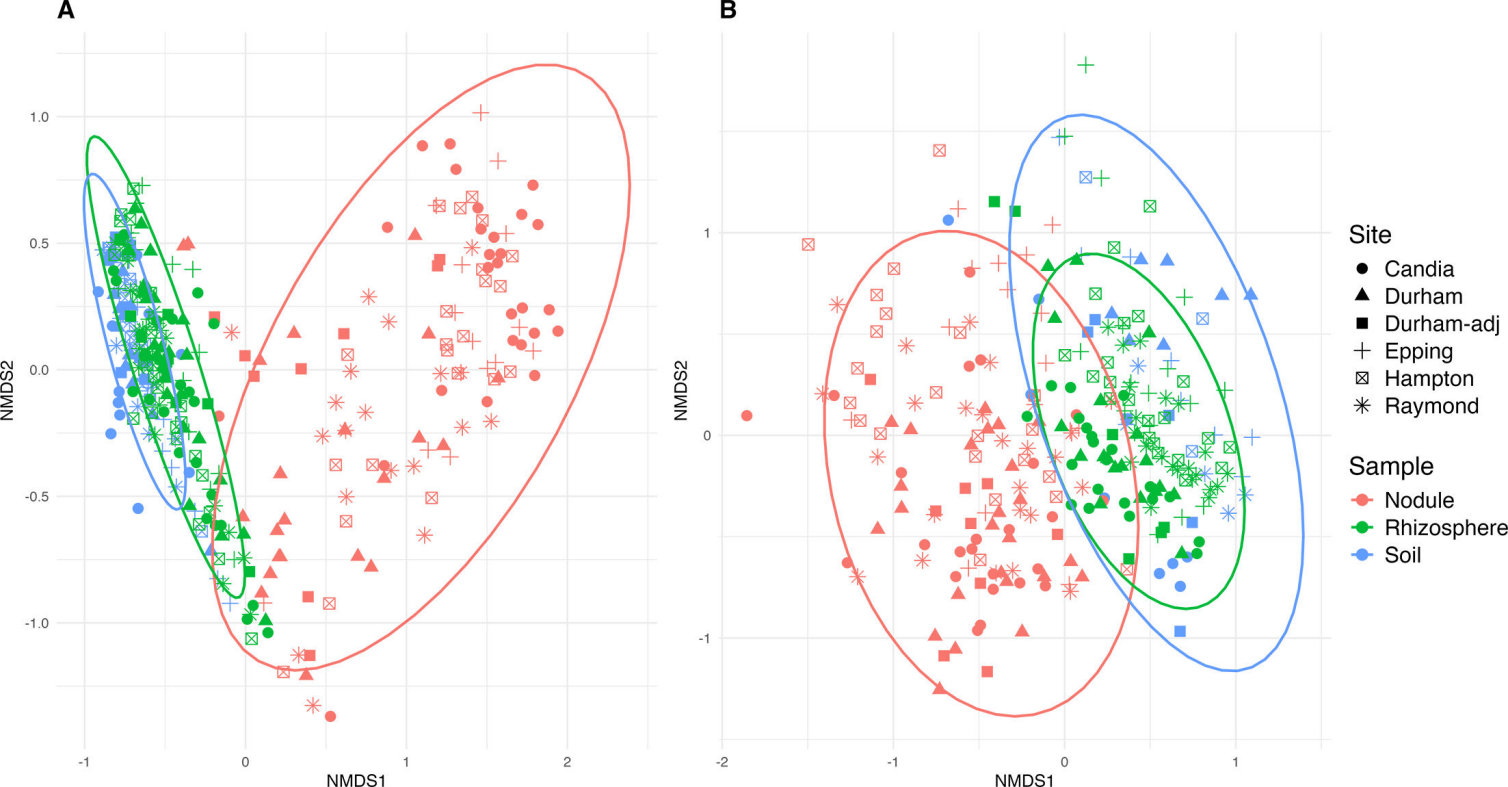
NMDS ordinations of bacterial (**A**, stress = 0.08) and fungal (**B**, stress = 0.17) communities. Ellipses were calculated with 95% confidence for the nodule (red ellipses), rhizosphere (green ellipses), and soil (blue ellipses) communities from all sites.

Community dissimilarities between sample types and sampling sites are visualized in Fig. 2. Dissimilarities between the sample types in both the bacterial (Fig. 2A) and fungal (Fig. 2B) NMDS ordinations are reflected in the PERMANOVA results in Table S3 and Table S4, respectively. Sample types were significantly different in both the bacterial (*P* = 0.0001) and fungal (*P* = 0.0001) data sets, explaining 36.2% and 12.2% of the communities’ variations, respectively, though we also report significant beta dispersion by sample type for both data sets.

**Fig 3:**
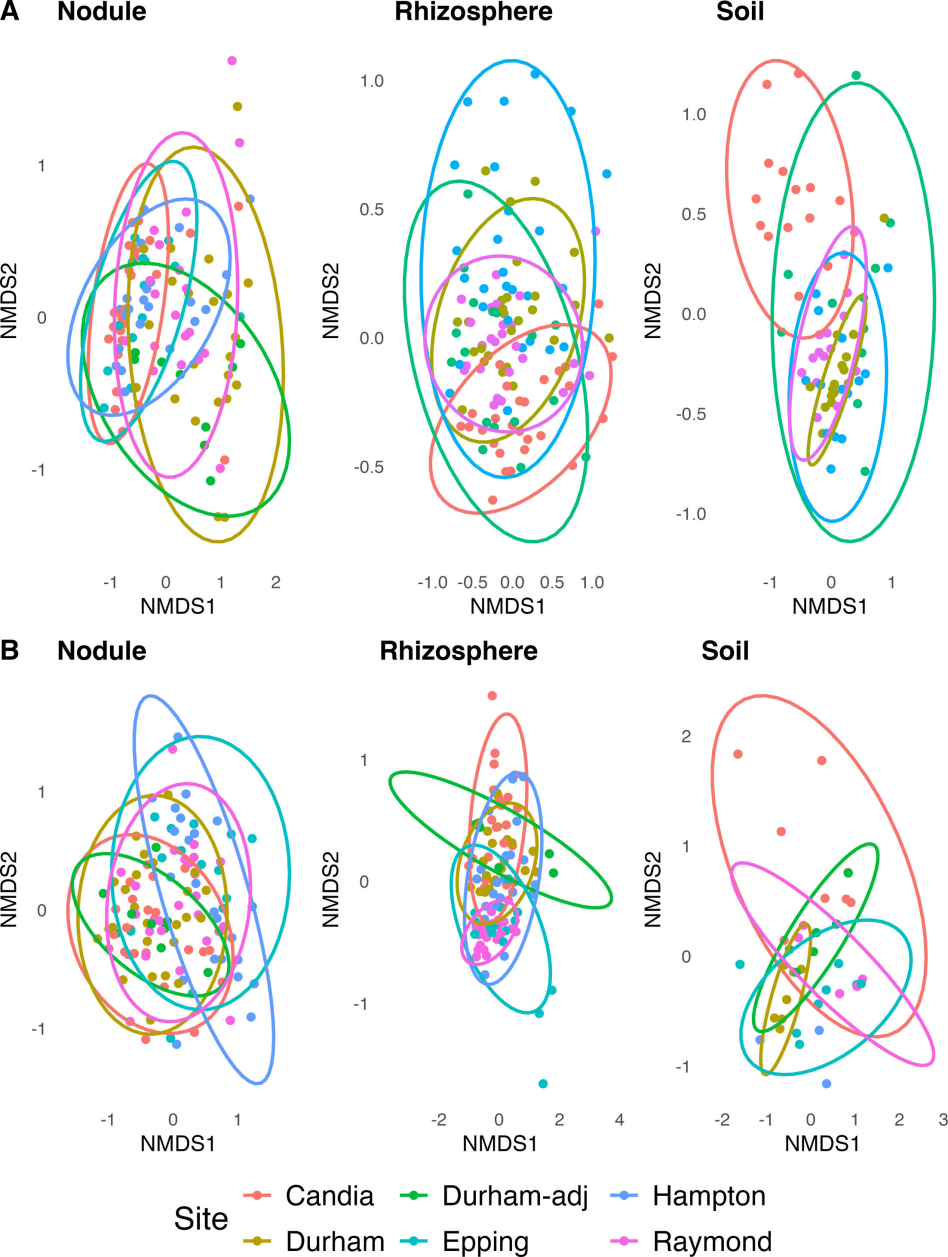
Individual NMDS ordinations for the bacterial (**A**) and fungal (**B**) nodule, rhizosphere, and bulk soil communities. Ellipses were calculated with 95% confidence for the Candia (red), Durham (brown), Durham-adjacent (green), Epping (teal), Hampton (blue), and Raymond (pink) communities. Too few points were present in the fungal soil communities to calculate an ellipse for the Hampton site. Stresses for the NMDS ordinations are as follows: bacterial nodule = 0.13; bacterial rhizosphere = 0.12; bacterial soil = 0.08; fungal nodule = 0.16; fungal rhizosphere = 0.14; fungal soil = 0.11.

Community dissimilarities by sampling site at the nodule, rhizosphere, and bulk soil levels can be seen for the bacterial and fungal communities in Fig. 3A and B, respectively. Sampling site explained 21.5% of the bacterial nodule community variation (*P* = 0.0001). The fungal nodule communities significantly differed by sampling site (*P =* 0.0001) with sampling site explaining 15.4% of the community variation though beta dispersion was significant for the fungal nodule communities by sampling site. Sampling site had a significant effect on the bacterial (*P* = 0.0001) and fungal (*P* = 0.0001) rhizosphere communities, explaining 14.9% and 18.7% of the community variation, respectively. The soil communities, which were the communities collected away from the host trees, were significantly affected by the sampling site which explained 36.7% of the bacterial community variation (*P* = 0.0001) and 25.9% of the fungal community variation (*P* = 0.0001).

### Occupants of the alder nodule microbiome

**Fig 4:**
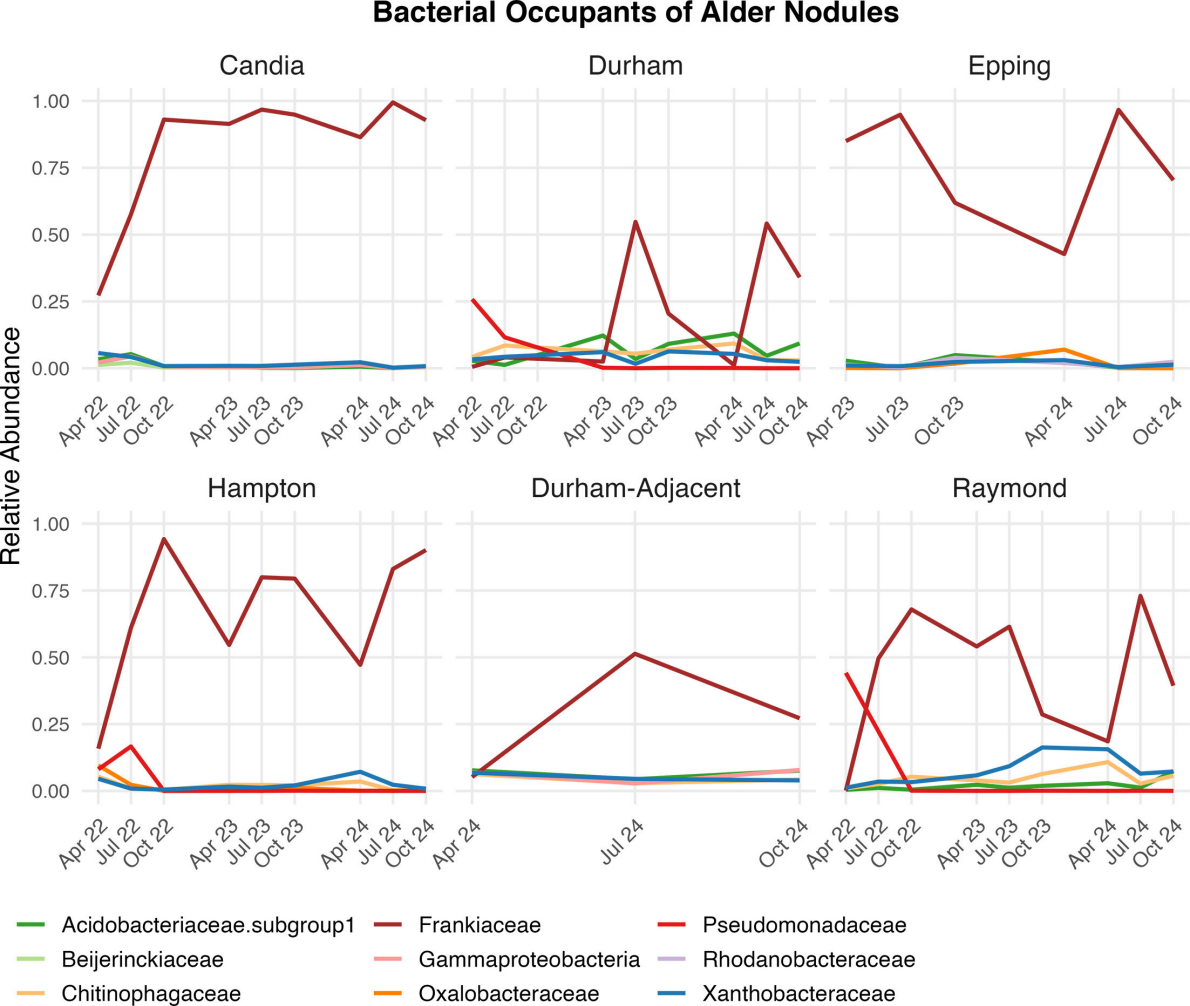
Seasonal dynamics in nodule occupants over the nine sampling periods for the bacterial alder nodule microbiomes. For simplicity, only the top five families with the highest relative abundance per site were plotted.

**Fig 5:**
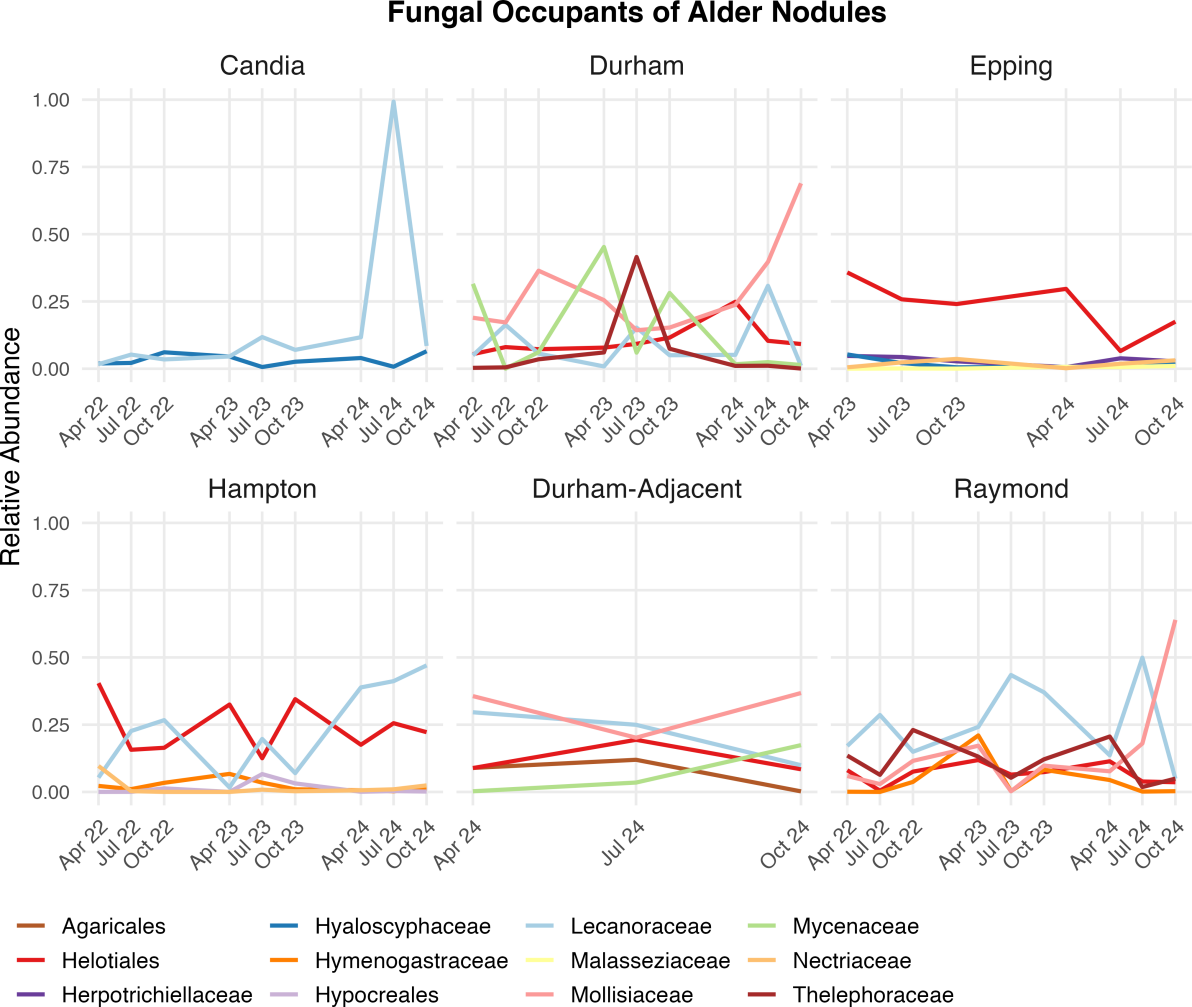
Seasonal dynamics in the fungal nodule occupants over the nine sampling periods. For simplicity, only the top five families with the highest relative abundance per site were plotted.

For visualizing the seasonal shifts in the alder nodule microbiome, we filtered the data set for only those taxa that were present in 100% of nodule samples per site across all seasons (April, July, and October). Then, the five nodule taxa with the highest total relative abundance per site in both the bacterial and fungal communities were plotted over all nine sampling periods in Fig. 4 and Fig. 5, respectively.

For the Candia, Epping, and Hampton sites, *Frankiaceae* were dominant nodule occupants at each sampling period. *Frankiaceae* occupancy showed cyclicity with peaks in July and often with lower occupancy levels in the April and October periods for most of the sites and sampling periods (Fig. 4). This pattern was not reflected in 2022 where all available sites had the highest *Frankiaceae* occupancy during October. The Hampton site exhibited different occupancy patterns with seasonal peaks of *Frankiaceae* every October. As *Frankiaceae* abundance peaks in July with the seasonal peak of photosynthesis, other nodule members often decrease. Certain fungal occupants like *Lecanoraceae* showed similar seasonal cyclicity, while others’ seasonal patterns were site dependent like *Thelephoraceae*, which decreased in July 2023 in Raymond but increased in July 2023 in Durham.

*Acidobacteriaceae* subgroup 1, *Xanthobacteraceae,* and *Chitinophagaceae* were other consistent occupants in the alder nodules (Fig. S7). The Candia rhizospheres had more consistent occupancy of *Acidobacteriaceae* subgroup 2, Gammaproteobacteria, and the Uncultured bacteria 7, compared to the roadway rhizospheres that had more *Chitinophagaceae* (Fig. S8). The Candia soils also showed distinct differences compared to the roadway soils with more Uncultured bacteria 7 and *Acidobacteriaceae* subgroup 1. The roadway bulk soils all had consistent occupancy of *Nitrosomonadaceae,* which was not a consistent occupant in the Candia soils (Fig. S9). All soils had consistent occupancy of *Pedosphaeraceae*.

For the fungal nodule occupants, Candia only had two consistent nodule fungal occupants that passed the filtering criteria. *Lecanoraceae* was a shared occupant among all sites’ nodules except Epping. All sites except Candia had consistent nodule occupancy of *Helotiales.* For the Durham and Durham-adjacent sites, fungal occupants from *Mollisiaceae* had higher relative abundances than *Frankiaceae* (Fig. S10). The Durham and Durham-adjacent sites both uniquely shared consistent nodule occupants from *Mycenaceae* and had more consistent fungal occupants than the other sites (Fig. S10). All the alder rhizospheres shared consistent *Thelephoraceae* occupants*,* with Raymond having the highest relative abundance, and *Helotiales* was a consistent occupant at five of the six sampling sites (Fig. S11). The bulk soil fungal occupants were more varied than the other sample types though *Thelephoraceae* was a consistent soil occupant at four of the sampling sites (Fig. S12).

### Soil influences the alder bacterial and fungal communities

**Fig 6:**
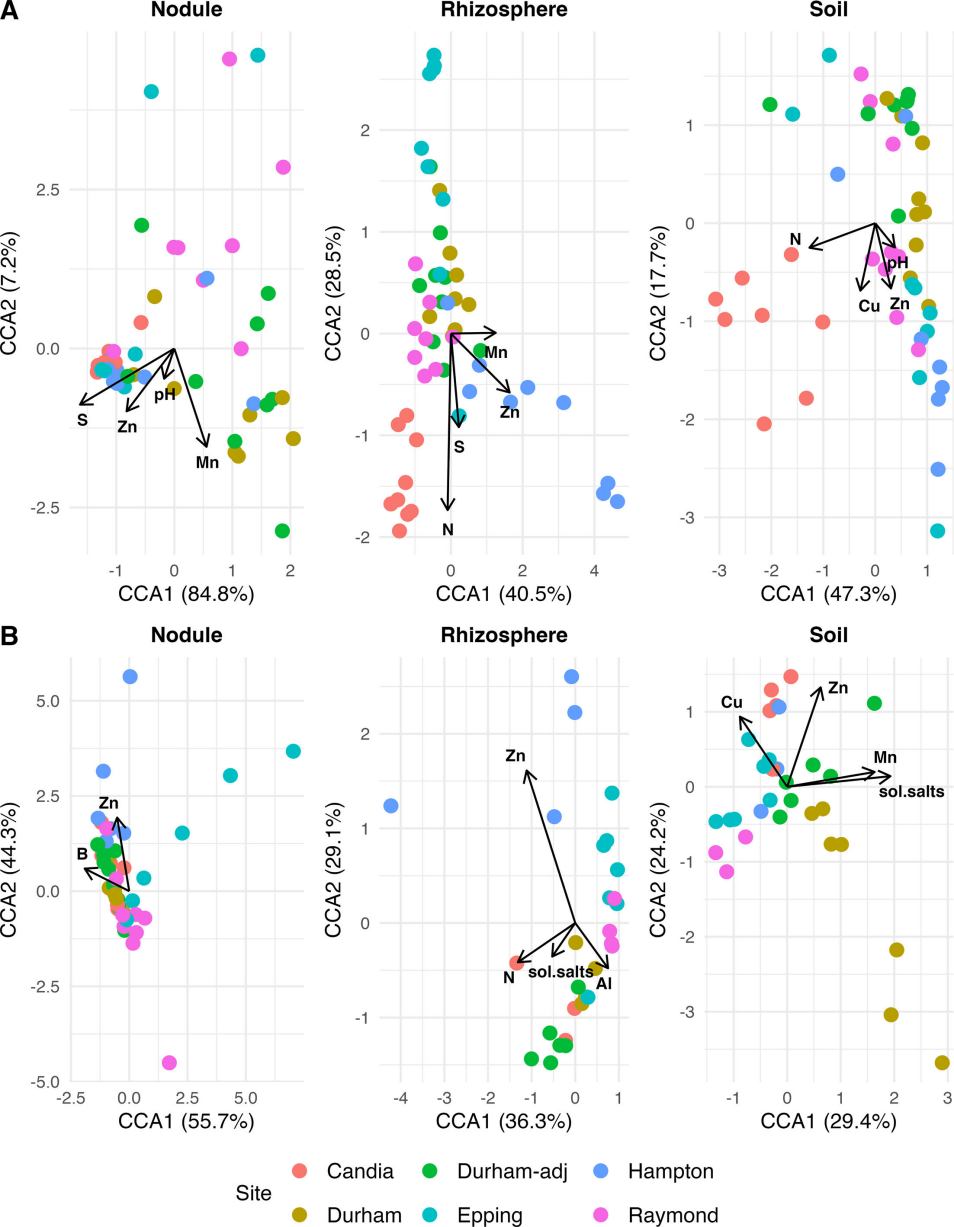
CCA of the 2024 bacterial (**A**) and fungal (**B**) communities constrained by soil variables.

Visualization of the soil variable influence on the bacterial and fungal communities’ structures was done using a canonical correspondence analysis (CCA) constrained by the measured soil variables (Fig. 6) (Table S5). For simplicity, only the top four statistically significant soil variables driving the community structures are plotted on the CCA. ANOVA results for all soil variables that passed the ordistep selection are in Table S6 for the bacterial communities and Table S7 for the fungal communities. Of the significant variables found via the CCA, Zn was a strong predictor of all the bacterial communities’ structures (Fig. 6A). S and Mn were significant predictors for all the bacterial communities as well (Table S6). The soil variables explained the most variation in the bacterial soil communities at 41.9%, followed by the bacterial rhizosphere at 31.9% and the bacterial nodule communities at 29% (Table S6).

For the fungal communities, Zn was, like the bacterial communities, a strong predictor of community structure for all fungal communities (Fig. 6B). The soil variables explained the most variation in the fungal rhizosphere communities at 18.6%, followed by the fungal bulk soil communities at 17.8% and the fungal nodules at 4.8% (Table S7). The soil variables explained less variation in the fungal communities than the bacterial communities. The fungal nodule communities only had B and Zn as significant predictors of community structure.

## DISCUSSION

### Regional droughts affect *Frankia* nodule occupancy

The Candia, Epping, and Hampton sites had the highest overall relative abundances of nodule *Frankiaceae* occupancy. Though different in their site characteristics, they had the most consistent water sources compared to the Raymond and Durham sites, and levels of soil moisture affect *Frankia* abundance and rates of nitrogen fixation in alder nodules (21). We have shown that varying levels of *Frankiaceae* members in actinorhizal nodules *in situ* at different sites and speculated that these differences could have been due to the varying levels of humidity that characterize the three sampling sites (7). Our current results support that speculation. Though soil moisture was not measured, this pattern is seen in the region-wide drought of the spring and summer of 2022 when the *Frankiaceae* relative abundance was lowest in all the available sites’ nodules in April and July and peaked in October. Typically, nitrogen fixation peaks in midsummer with available photosynthate supply (17, 22, 23) and as supported in Fig. 4 for years 2023 and 2024. With less carbon sources being allocated to the nodules under drought stress, *Frankia* relative abundance would be lower in root nodules due to the expense of nitrogen fixation under insufficient soil moisture levels (21). A relationship between nodule nitrogen fixation and soil moisture in actinorhizal plants and legumes has been shown (24–26).

### Alder nodules harbor different relative abundances of *Frankia*

Under different environmental stressors, the actinorhizal system can maintain nodulation and nitrogen fixation (1, 2, 27). However, community analyses have reported varying relative abundances of *Frankia* in both *Casuarina* and *Alnus* nodules (7, 28–30). Though the scope of this study does not allow for the determination of the cause of the differing abundances of *Frankiaceae* in nodules reported here, we have some speculations. For example, The Durham and Durham-adjacent nodule communities had the lowest *Frankiaceae* relative abundance and the highest relative abundance of fungal nodule occupants, especially *Mollisiaceae* and *Mycenaceae,* indicating possible competition between the present and potential nodule occupants. Though we do not know the order of infection, the priority effects of one root endophyte could hinder successive attempts at root colonization by other endophytes, especially since ECM presence negatively impacts *Frankia* nodulation of *Alnus* (31, 32). Additionally, *in vitro* competition assays with *Frankia* and other, culturable bacterial nodule inhabitants resulted in positive, neutral, and negative interactions between the symbiont (33). If a non-*Frankia* nodule occupant is inhibitory toward *Frankia* colonization, the efficiency of the actinorhizal symbiosis will be affected due to the lower abundances of Frankia and the lowered rates of nitrogen fixation. Future work involving nodule occupancy needs to assess how actinorhizal plants are affected by differing abundances of *Frankia* in nodules and how other microbes may affect *Frankia* colonization.

### Rural alder nodules and soils are less diverse than those from roadways

The rural Candia rhizosphere, soil, and most of the nodule bacterial communities had significantly lower alpha diversities than the roadway sites. It was expected that this stark distinction between the rural and roadway sites would be reflected in the fungal communities as well since fungal communities decrease in diversity in roadside soils, but this was not reported here (20). However, increases in roadside bacterial diversity, which we do report, have been reported previously (20). Roadways present many challenges to the adjacent soil and plant communities such as increases of metals (34, 35), and Zn was a significant predictor for all microbial communities (Table S6 and S7). These differences could also be attributed to the rural sites’ high levels of soil Al and S, a characteristic of the peatlands at this site (Table S5) (36, 37). The selective effects of Al and S are likely additional drivers of the relatively low bacterial alpha diversity at the Candia site (38–40). The Candia site, with its dominance of *Frankiacecae* in nodules, consistently had the highest levels of soil total N in 2024, but the Epping site, which had similarly high abundances of *Frankiaceae*, had the lowest levels of soil total N in July and October of 2024 and the second lowest soil total N in April of that same year (Table S5). Additional studies need to address the effect of different soil conditions on the colonization and persistence of actinorhizal-associated bacterial and fungal occupants.

### Alders share root, rhizosphere, and soil microbes with other plant hosts

The bacterial community members reported here are not unique to this study. The *A. glutinosa* root endosphere and rhizosphere contain *Acidobacteriota* and *Burkholderiales*, and in *Hippophae tibetana,* another actinorhizal plant, rhizosphere communities harbor members of *Chitinophagaceae* and *Micropepsaceae* (11, 41). Outside of the actinorhizal symbiosis, there are also reports of *Pedosphaeraceae* members in poplar roots and *Opitutaceae* members in the maize rhizosphere *in situ* (42, 43). Additionally, *Nitrosomonadaceae*, which was a consistent occupant of all roadside soils (Fig. S9), is dominant occupant of roadside ditches (44, 45).

Previous studies also reported several of the alder-associated fungal occupants found in this study. Members of *Thelephoraceae, Mollisiaceae*, and *Russulaceae* in *A. glutinosa* roots and salinity and heavy metals influenced levels of some alder ECM symbiont taxa like *Thelephoraceae* and *Helotiales* in alder, goat willow, and silver birch (46–48). The Raymond rhizospheres had the highest consistent levels of *Thelephoraceae*, which also boasted the highest levels of Fe in July and October though more investigation into this possible *in situ* connection is needed since the Raymond site had the lowest levels of Fe in April (Table S5). It is also possible that these trees were utilizing ECM associations for increased water uptake in this drier roadside site (5). Future metagenomic work will improve the taxonomic resolution of these reported communities in order to corroborate the presence of non-*Frankia* plant-growth-promoting bacteria and mycorrhizal fungi, as well as the presence of metal and polycyclic aromatic hydrocarbon resistant organisms in the roadside samples (20).

### Conclusion

We report the influence of season, location, and roadsides on the *A. rugosa* nodule and rhizosphere microbiome. Though this field study allows a glance into the *in situ* microbial world of the *A. rugosa* root and soil microbiomes, it is important to note that certain environmental variables were not measured due to the scope of this study. Future field studies of the actinorhizal symbiosis should include whole genome sequencing of the host plants as an effect of host phylogeny on the acquired *Frankia* community was found (49). Those soil variables that showed influence on these measured communities should be validated in controlled greenhouse experiments to confirm their effects on the actinorhizal relationship with hosts of a confirmed age and genotype. Additionally, metagenomic sequencing would offer valuable insights into the functional potential of these wild actinorhizal nodule communities. There is still a lack of studies reporting shifts in the associated *Alnus* microbiomes over longer periods of time in different locations. Understanding how these pioneer plants’ microbiomes are changing over time *in situ* and in relation to environmental disturbances and contaminants is imperative for successful and lasting applications of the actinorhizal symbiosis in partner-planting and bioremediation efforts.

## MATERIALS AND METHODS

### Sampling sites

**Fig 7:**
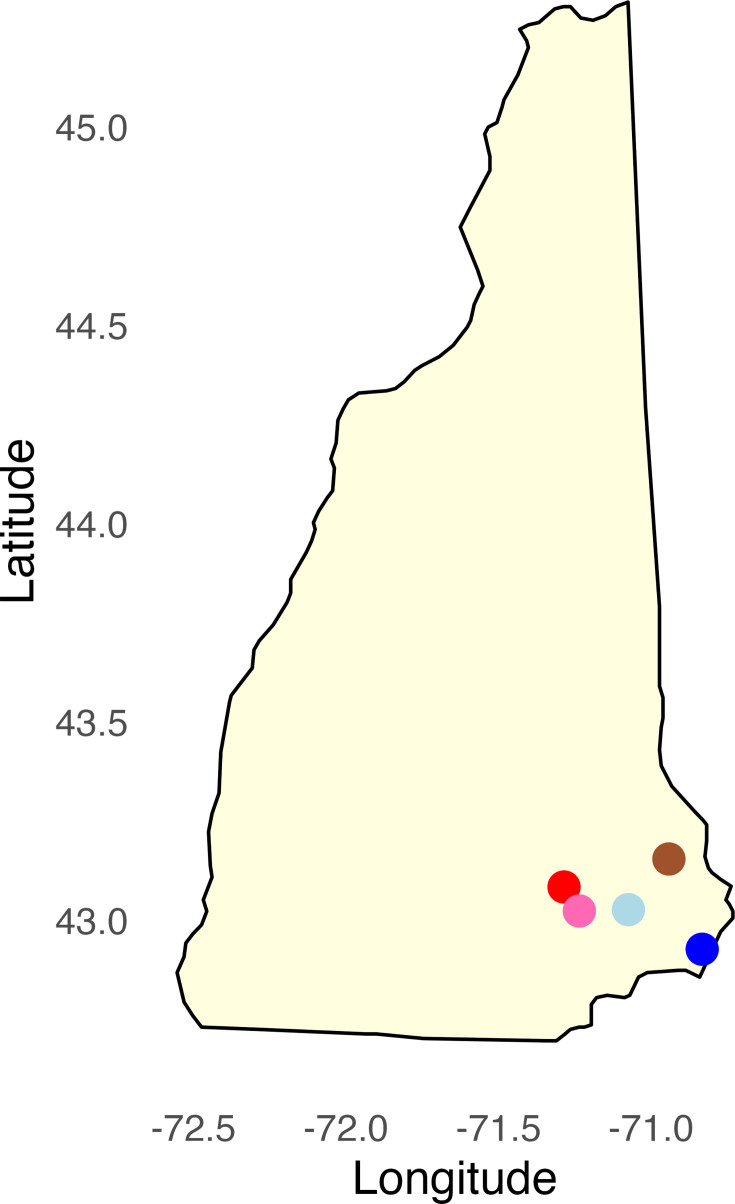
Map of New Hampshire with sampling sites. Red indicates the Candia site, pink indicates the Raymond site, light blue indicates the Epping site, dark blue indicates the Hampton site, and brown represents the Durham and Durham-adjacent sites.

Six sites located in southeastern New Hampshire were chosen for this study based on their habitat characteristics and proximity to major roadways (Fig. 7). Site 1 is in Candia, New Hampshire in the Deerfield Road Town Parcel (43.08,455 N 71.28,530 W), managed by the Candia Conservation Committee. These trees are located on the border of a freshwater wetland, about 1.6–2.4 km from the nearest roadway. Site 2 is in Raymond, New Hampshire (43.02,402 N 71.23,504 W), on a strip of highway land without a permanent water source. The trees are about 15 m from State-Route 101, a major state highway. Site 3 is in Hampton, New Hampshire (42.92,760 N 70.83,230 W), where the trees are about 15 m from State-Route 101 and adjacent to the local salt-water marsh. Site 4 is in Epping, New Hampshire (43.027,438 N 71.074,194 W), where the trees are on a small patch of land between a fast-food parking lot and the ramp for State-Route 101. Most of the water to Site 4 is provided by a drainage ditch. The Epping site was added in 2023 for its urbanized location. Site 5 is in Durham, New Hampshire (43.15,510 N 70.94,141 W), where the trees are located about 23 m from Route 4 and border a freshwater wetland. Site 6, Durham-adjacent, was added in 2024 to compare to the original Durham site. The Durham-adjacent site is located about 91 m from the Durham site in a more wooded area about 30 m from Route 4 (43.15495 N 70.94,226 W).

For sampling, the same three trees from each site were used for nodule collection each sampling period unless there was damage or removal of trees. At the Epping site, two of the original Epping trees were cut in the late summer of 2023, so two new alder trees from the same site were sampled from October 2023 through 2024. At the Hampton site, much of the sampling area was cleared over the winter of 2023–2024, so three of the remaining alders were sampled for all of 2024. Otherwise, all original trees were sampled through the duration of the sampling project.

### Sample collection and processing

Each site was sampled in April, July, and October in 2022, 2023, and 2024, with the same trees being sampled from throughout the duration of the study. Approximately 10 g of mature nodules was collected from each tree, and each tree was tagged for future identification. Bulk soil (50 g) was collected from a depth of 15 cm in sterile sampling bags at each site, with care being taken to not collect soil near alder rhizospheres. Sample collection was completed using sterile sampling bags and gloved hands. Nodules and soils were frozen at −20℃ until DNA extraction.

For rhizosphere collection, nodules were placed in sterile tubes with 8 mL of sterile distilled water and vortexed on the highest setting for 1 min. The rinsed nodules were removed from the tubes and set aside for surface sterilization and DNA extraction. The remaining solution contained the rhizosphere closely associated with the plants. The washed rhizosphere was centrifuged at 4,300 × *g* for 20 min. The supernatant was removed. The washed rhizosphere was collected and stored at −20℃ until DNA extraction.

To prepare for the nodule DNA extraction protocol, nodules were rinsed with sterile water 5–7 times and vigorous shaking in tubes until the water was cleared of visible dirt. Nodules were surface sterilized by incubating in 30% hydrogen peroxide for 45 min with occasional mixing. After incubation, the treated nodules were washed with sterile water to remove the hydrogen peroxide. Once sterilized and rinsed, ~0.5 g nodules per tree were crushed using a sterilized mortar and pestle and liquid nitrogen. The resulting powder was collected and extracted as previously described, with some modifications (50). The nodule powder was incubated at 65℃ for 30 min in extraction buffer (100 mM Tris-HCl, pH 8; 20 mM EDTA pH 8.2; 1.4 M NaCl, 2% cetyltrimethylammonium bromide [CTAB]), followed by extraction with phenol-chloroform-isoamyl alcohol (25:24:1), and precipitated with ethanol, and the resulting DNA pellet was resuspended in nuclease free water. For the washed rhizosphere and bulk soils, DNA was extracted using the commercially available Qiagen PowerSoil Pro Kit (Qiagen, Hilden, Germany) using 0.5 g of material per sample, following the manufacturer’s protocol. All samples were RNAse treated using the Qiagen RNAse A enzyme following manufacturer’s instructions.

### Soil chemical analyses

To assess soil chemistry per sampling site, 100 g of soil was collected in triplicate in sterile sampling bags from each sampling site in 2024. Soil for chemical analysis was sent to the Cornell Soil Health Laboratory for their total carbon (total C) and total nitrogen (total N) test, soluble salts test, and their soil nutrient analysis package which included pH, percent organic matter, and values of plant available P, K, Ca, Mg, S, Al, Cu, Fe, Mn, Zn, and B (Cornell Soil Health Laboratory, Ithaca, NY, United States). All soil variables, excluding pH, were transformed using the log function in Microsoft Excel (v.16.54) prior to analysis and are available in Table S5.

### Amplicon processing and sequencing

Microbial community profiles for plant root nodules and soils were constructed for the prokaryotic and fungal members. For prokaryotes, the V4 and V5 hypervariable regions of the 16S subunit of the rRNA gene were used. The products were amplified and sequenced using the Earth Microbiome Project 515F/926R primers as described previously (51–53). For the fungal profiles, the internal transcribed spacer (ITS) region 2 of the rRNA genes was used. The product was amplified with the 5.8SF/ITS4R primer set and the ITS amplicons were sequenced (54).

Paired-end sequencing of the amplification products was completed using the Illumina NovaSeq 6000 platform (Illumina, San Diego, CA). Library preparation and sequencing was conducted by the Hubbard Center for Genome Studies at the University of New Hampshire (University of New Hampshire, Durham, NH, United States).

### Data processing and analysis

Amplicons were imported into the Quantitative Insights into Microbial Ecology 2 platform (v.2024.10) for processing and downstream analysis (55). Cutadapt (v. 2024.10) was used to trim primers from reads (56). The paired-end reads were denoised and dereplicated, and chimeras were removed using DADA2 (v. 2024.10) with the forward reads truncated to 232 bp and reverse reads truncated to 231 bp (57). Following denoising, a total of 59,515,344 16S rRNA gene amplicon sequence variants (ASVs) were retained and a total of 31,278,531 ITS ASVs were retained. The SILVA 138 database was used for training a naive Bayes classifier (28). The trained classifier was used for assigning taxonomy to reads (29). A pre-trained UNITE database (v. 10) was used for assigning taxonomy to ITS reads (30). All reads with taxonomic assignments to chloroplast, mitochondria, *Eukaryota* (16S only), or unassigned were removed from the data. Prior to statistical analyses, samples with low sequencing depth were removed (<20,000 reads for the 16S and <4,000 reads for the ITS data sets).

### Community and statistical analyses

The vegan package (v. 2.6-4) in R studio (4.2.1) was used for calculating Shannon’s diversity index for alpha diversity using the diversity function, bray-curtis matrix, and subsequent non-metric multidimensional scaling (NMDS) ordination creation using vegdist and metaMDS functions, respectively, and for the adonis2 function for PERMANOVA analysis for testing for community dissimilarity using 9,999 permutations (58–60). The betadisper function, also from the vegan package, was used for testing multivariate homogeneity of variances. Shannon diversity indices were calculated on filtered data sets subsampled to the 20,000 reads for the 16S datatset and to 4,000 reads for the ITS data set. Alpha rarefaction curves for the 16S and ITS data set were calculated using the rarecurve function in the vegan package (58) (Fig. S13). NMDS ordinations and PERMANOVAs were calculated using Bray-Curtis matrices calculated from the filtered data sets. Influence of soil variables on the 2024 microbial community structure was evaluated using canonical correspondence analysis (CCA) using the cca function from the vegan package (58). The ANOVA.cca function was used for calculating significance of constraints using 999 permutations. Multicollinearity was assessed among the soil variables by calculating the variance inflation factors using the vif.cca function from the vegan package (58). Variables with a VIF greater than 10 were omitted from the downstream CCAs to avoid redundancy and unstable results due to CCA sensitivity to multicollinear variables. The remaining soil variables were selected for the CCA using the ordistep function from the vegan package using both forward and backward stepwise selection for the CCA (58). For simplicity, only the top four statistically significant soil variables driving the community structures are plotted on the CCA. ANOVA results for all soil variables that passed the ordistep selection are in Table S6 for the bacterial communities and Table S7 for the fungal communities. Kruskal-Wallis rank sum test was calculated to test for significant differences between Shannon diversity indices between sites, followed by the Dunn’s post-hoc test if the Kruskal-Wallis results were significant to test for significance and magnitude of difference between sites using the dunn.test function in the dunn.test package (v. 1.3.6) (61–63). Ggplot2 was used for creation of graphs and ordination visualizations (3.5.2) (64).

## Data Availability

All sequence data (16S rRNA gene and ITS gene data sets) presented in this article are available in the repository of NCBI under BioProject numbers PRJNA1257386 and PRJNA1256894.

## SUPPLEMENTAL MATERIAL

Supplemental figures aem.00446-26-s0001.pdf Fig. S1 to S13.

Supplemental tables aem.00446-26-s0002.xlsx Tables S1 to S7.

ASM does not own the copyrights to Supplemental Material that may be linked to, or accessed through, an article. The authors have granted ASM a non-exclusive, world-wide license to publish the Supplemental Material files. Please contact the corresponding author directly for reuse.
