# Supplementary material for: A longitudinal roadside study of the New Hampshire alder root nodule microbiome
Source: Appl Environ Microbiol. 2026 May 27;92(6):e00446-26. doi: 10.1128/aem.00446-26 (PMC13274451; doi:10.1128/aem.00446-26)
Supplement: Supplemental figures — Fig. S1 to S13. [file aem.00446-26-s0001.pdf]

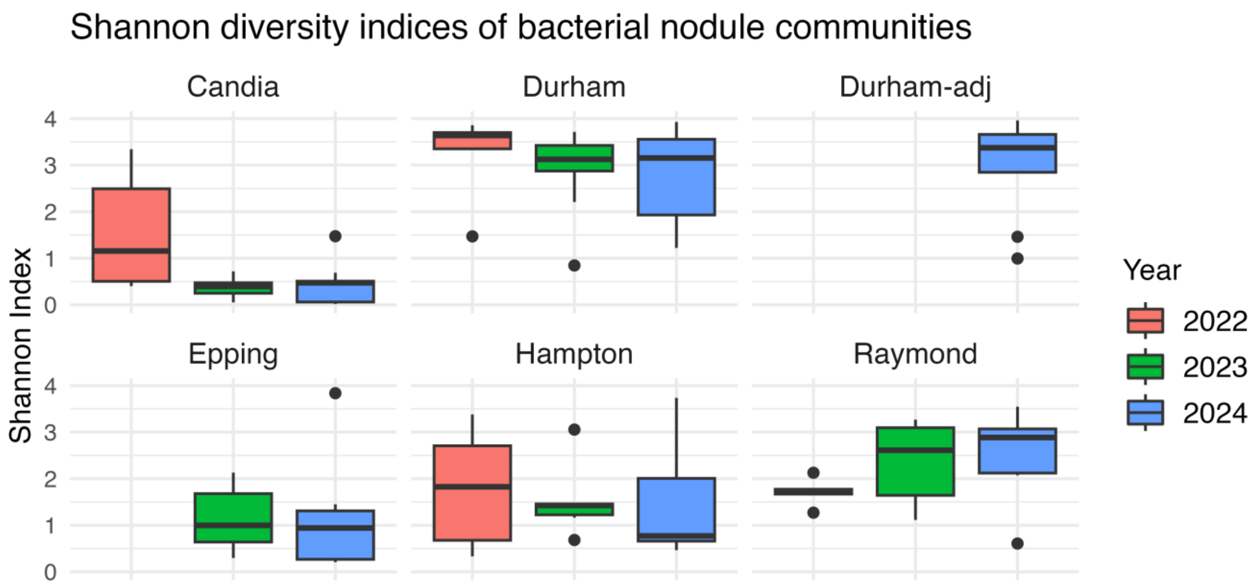

Figure S1. Shannon diversity index of the bacterial nodule communities at each sampling site and each sampling year. Colors denote the different sampling years.

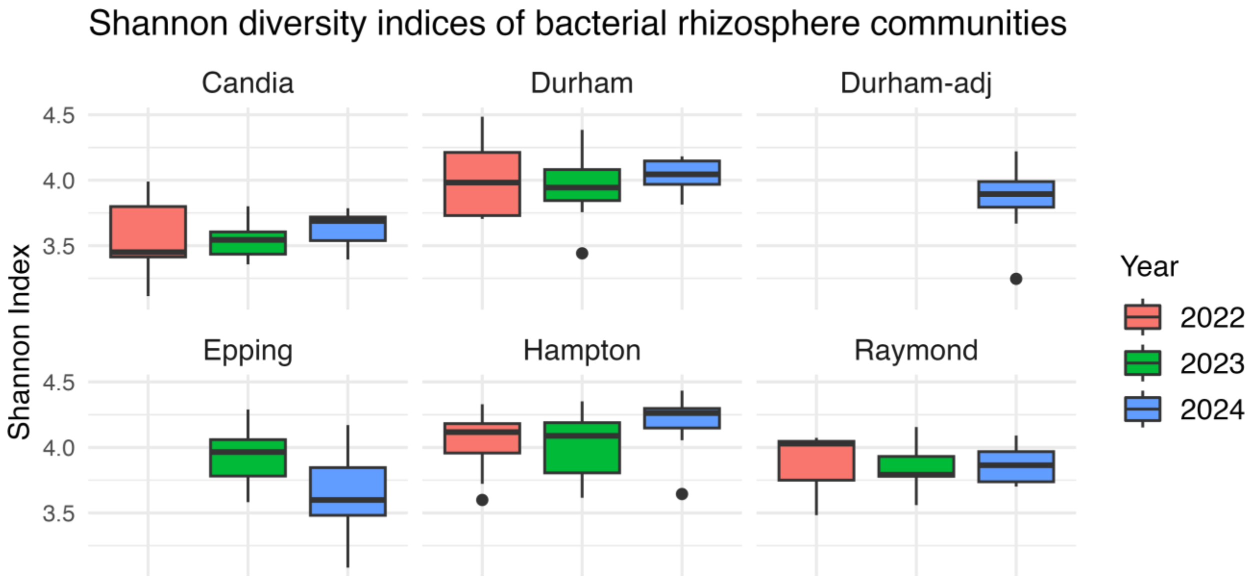

Figure S2. Shannon diversity index of the bacterial rhizosphere communities at each sampling site and each sampling year. Colors denote the different sampling years.

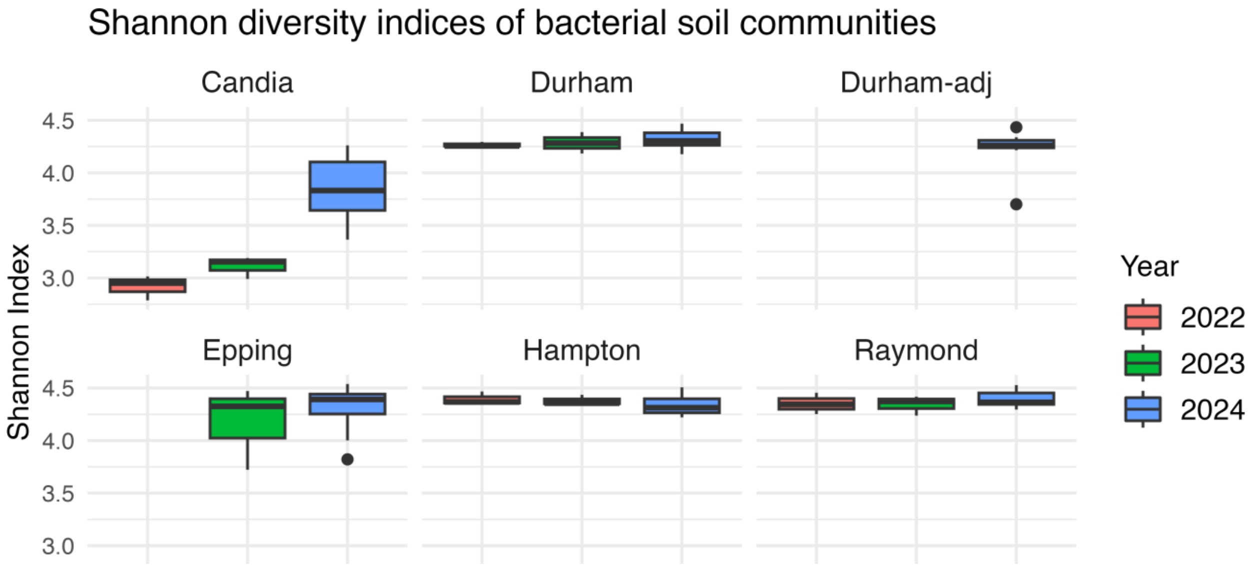

Figure S3. Shannon diversity index of the bacterial soil communities at each sampling site and each sampling year. Colors denote the different sampling years.

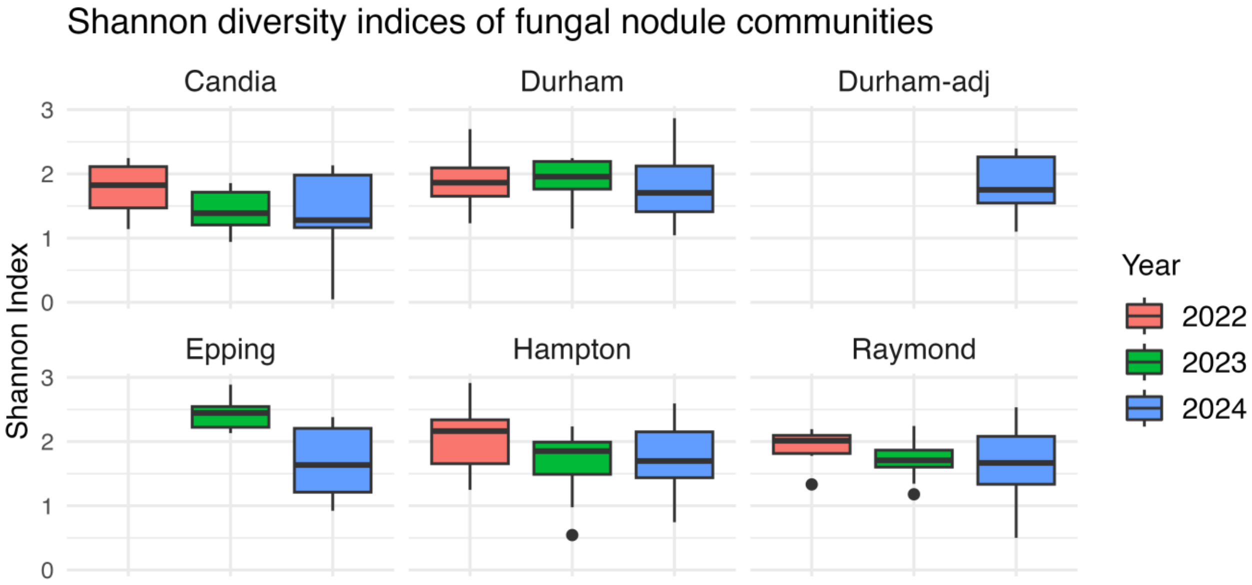

Figure S4. Shannon diversity index of the fungal nodule communities at each sampling site and each sampling year. Colors denote the different sampling years.

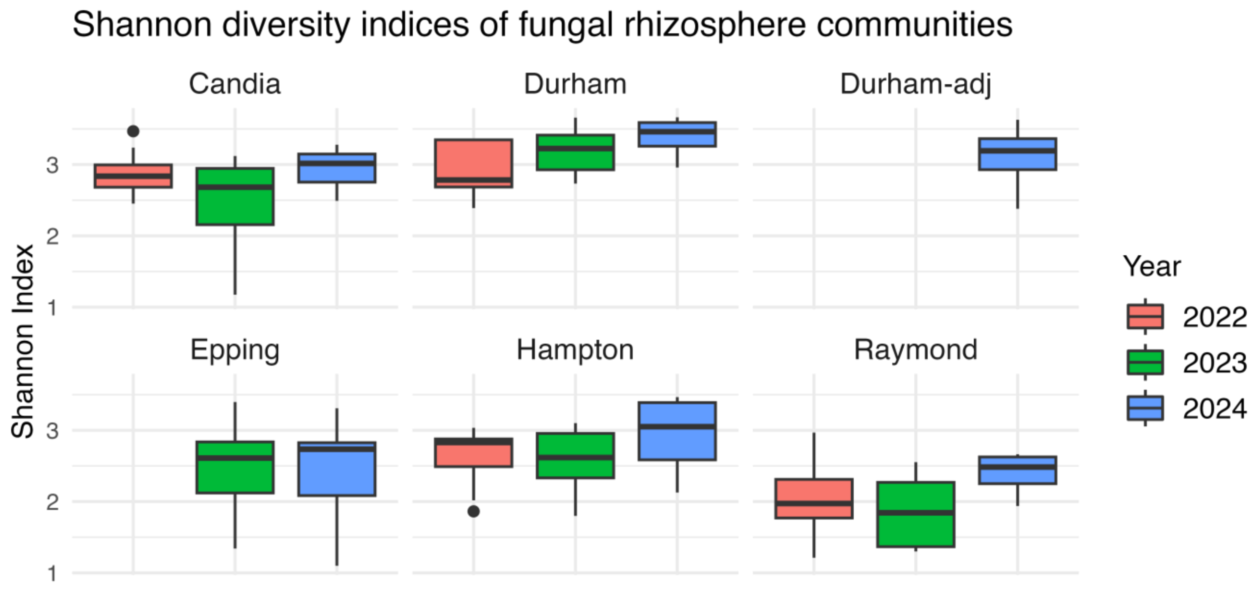

Figure S5. Shannon diversity index of the fungal rhizosphere communities at each sampling site and each sampling year. Colors denote the different sampling years.

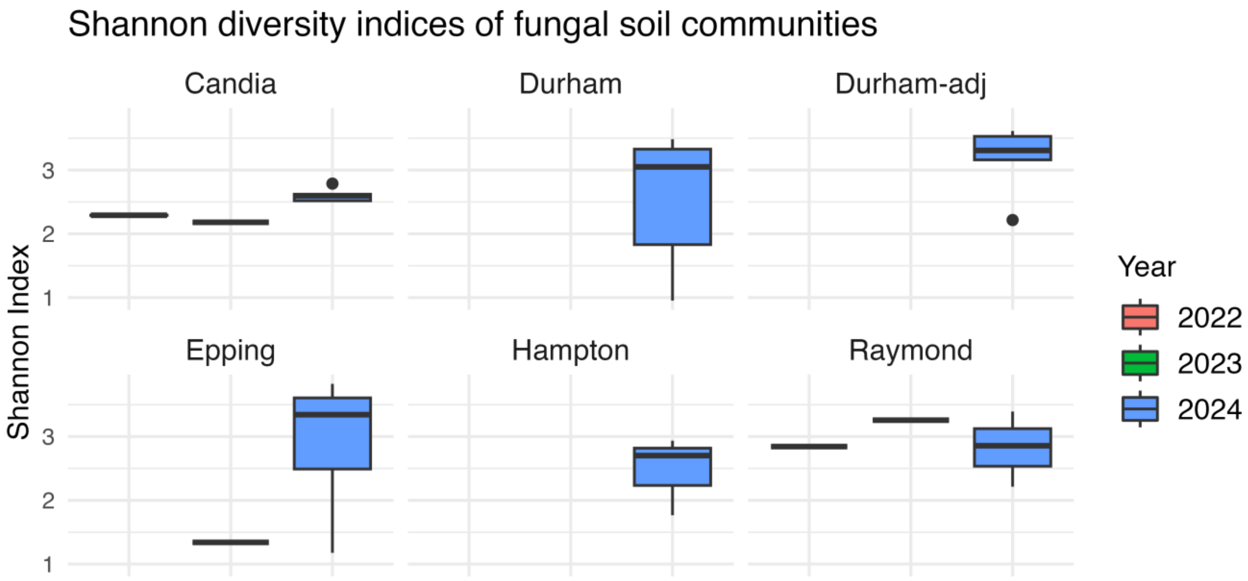

Figure S6. Shannon diversity index of the fungal soil communities at each sampling site and each sampling year. Colors denote the different sampling years.

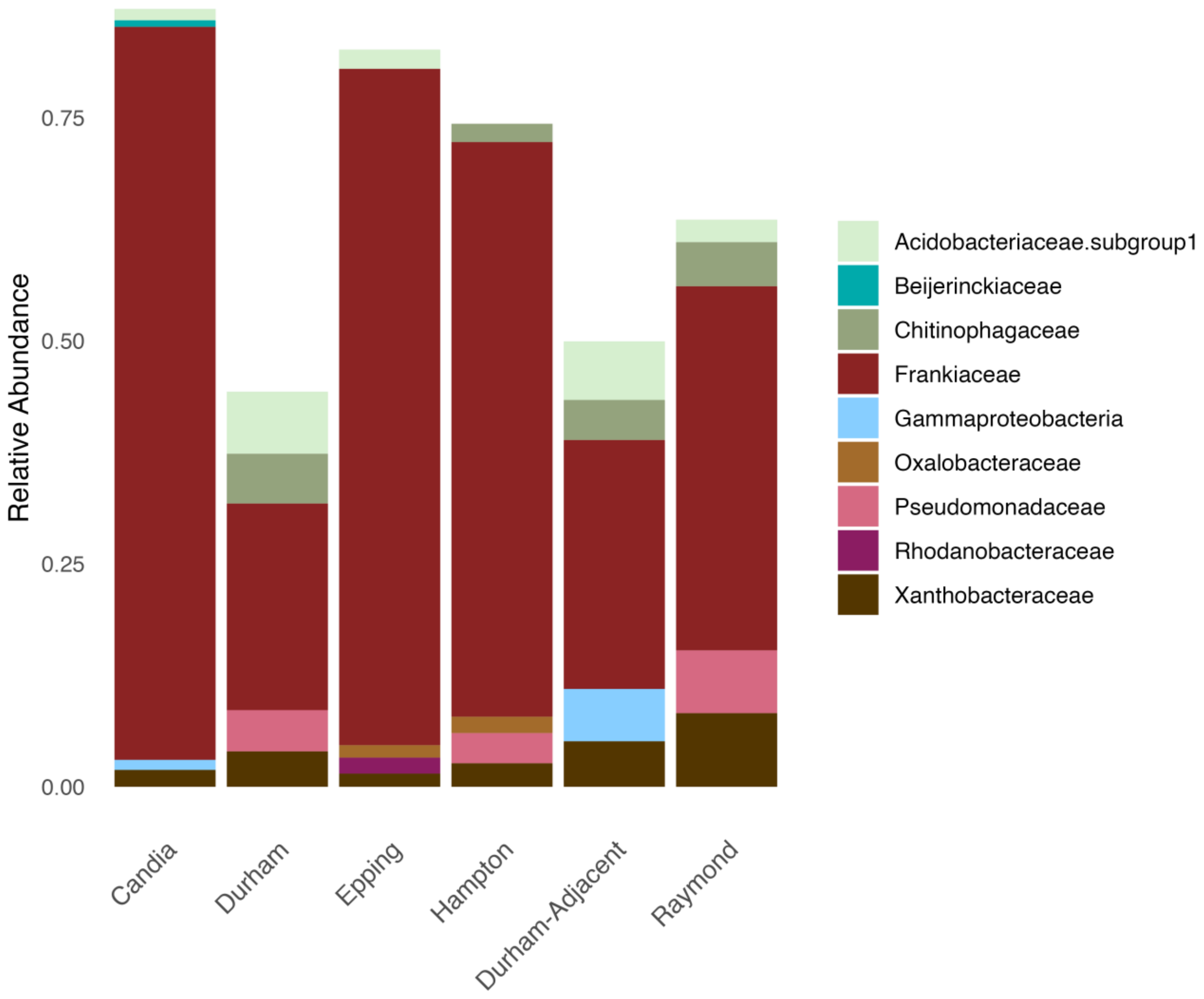

Figure S7. The top five bacterial taxa with the highest average relative abundance in the six sampling sites' alder nodules. Only those nodule occupants that were consistently found in 100% of nodule samples for each site were considered, then the top five bacterial taxa were plotted for simplicity.

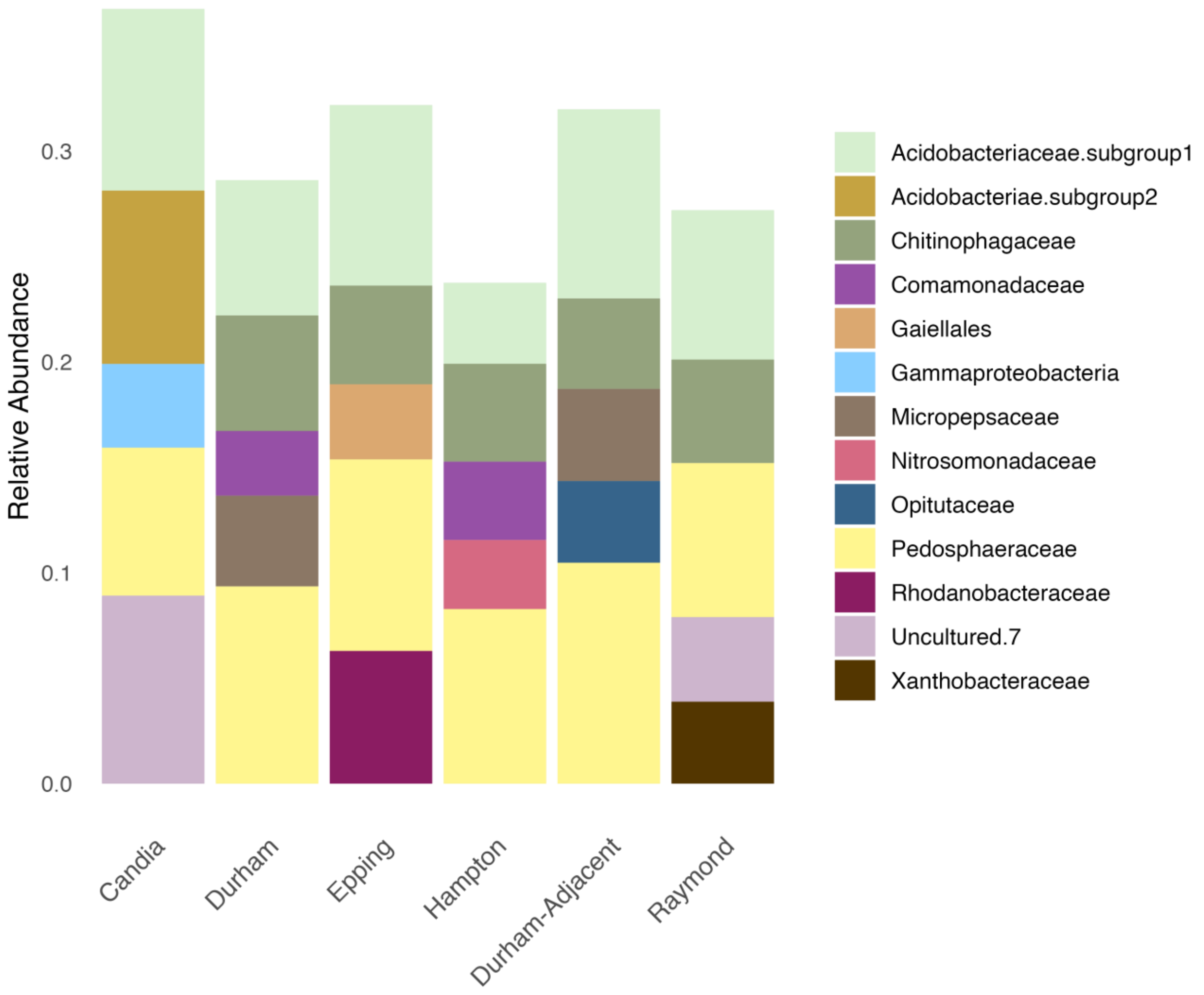

Figure S8. The top five bacterial taxa with the highest average relative abundance in the six sampling sites' alder rhizospheres. Only those rhizosphere occupants that were consistently found in 100% of rhizosphere samples for each site were considered, then the top five bacterial taxa were plotted for simplicity.

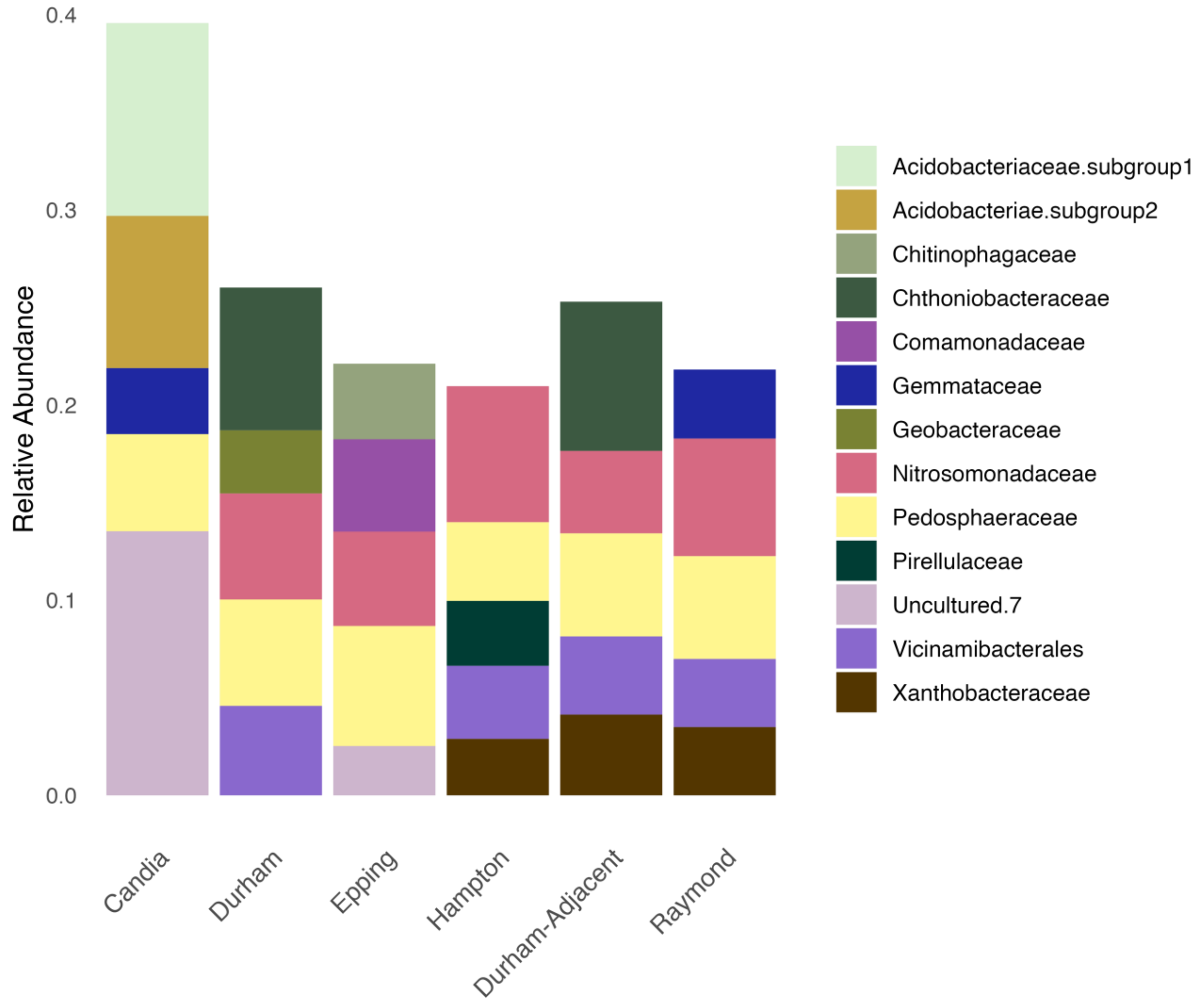

Figure S9. The top five bacterial taxa with the highest average relative abundance in the six sampling sites' bulk soils. Only those soil occupants that were consistently found in 100% of soil samples for each site were considered, then the top five bacterial taxa were plotted for simplicity.

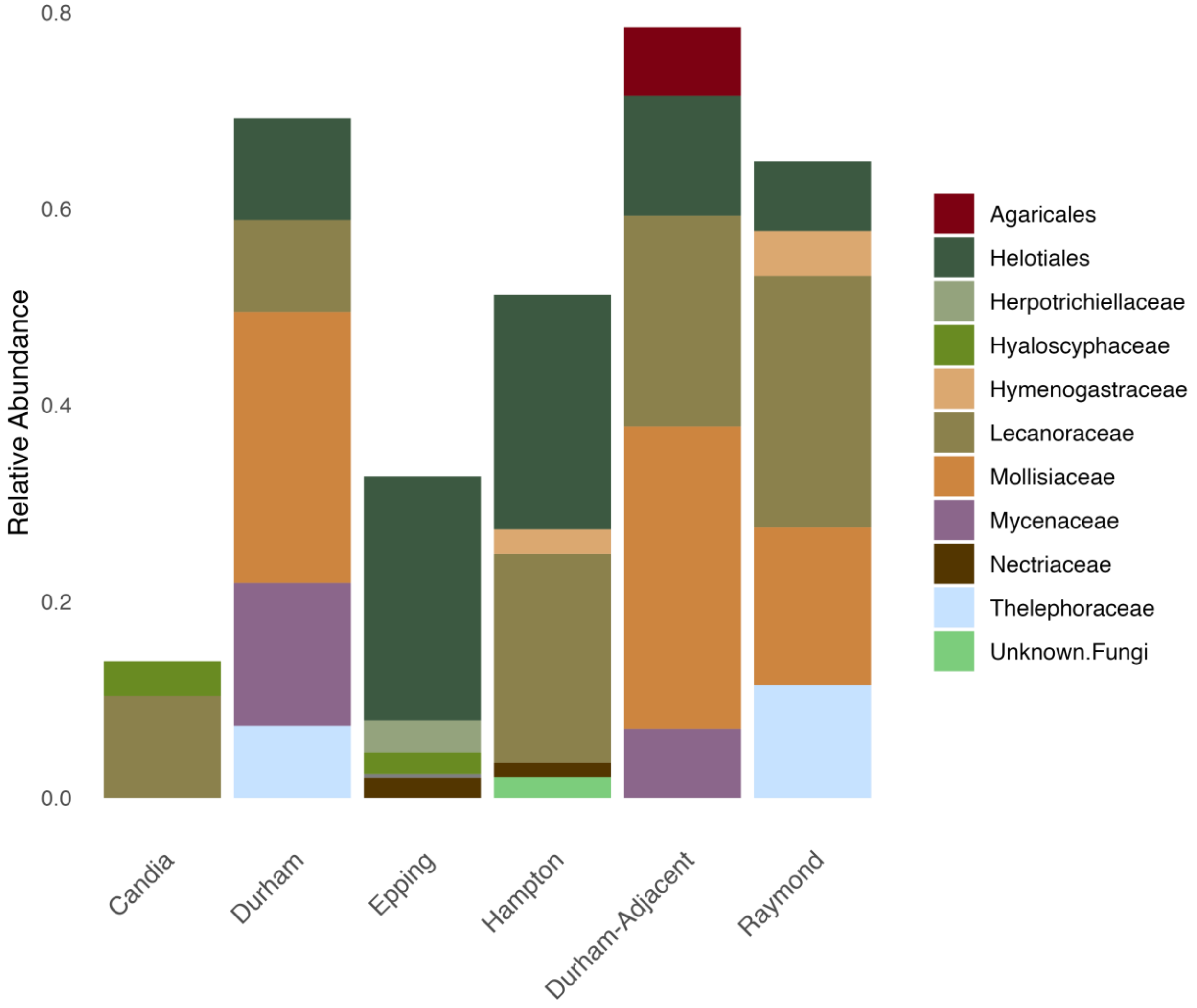

Figure S10. The top five fungal taxa with the highest average relative abundance in the six sampling sites' alder nodules. Only those nodule occupants that were consistently found in 100% of nodule samples for each site were considered, then the top five fungal taxa were plotted for simplicity.

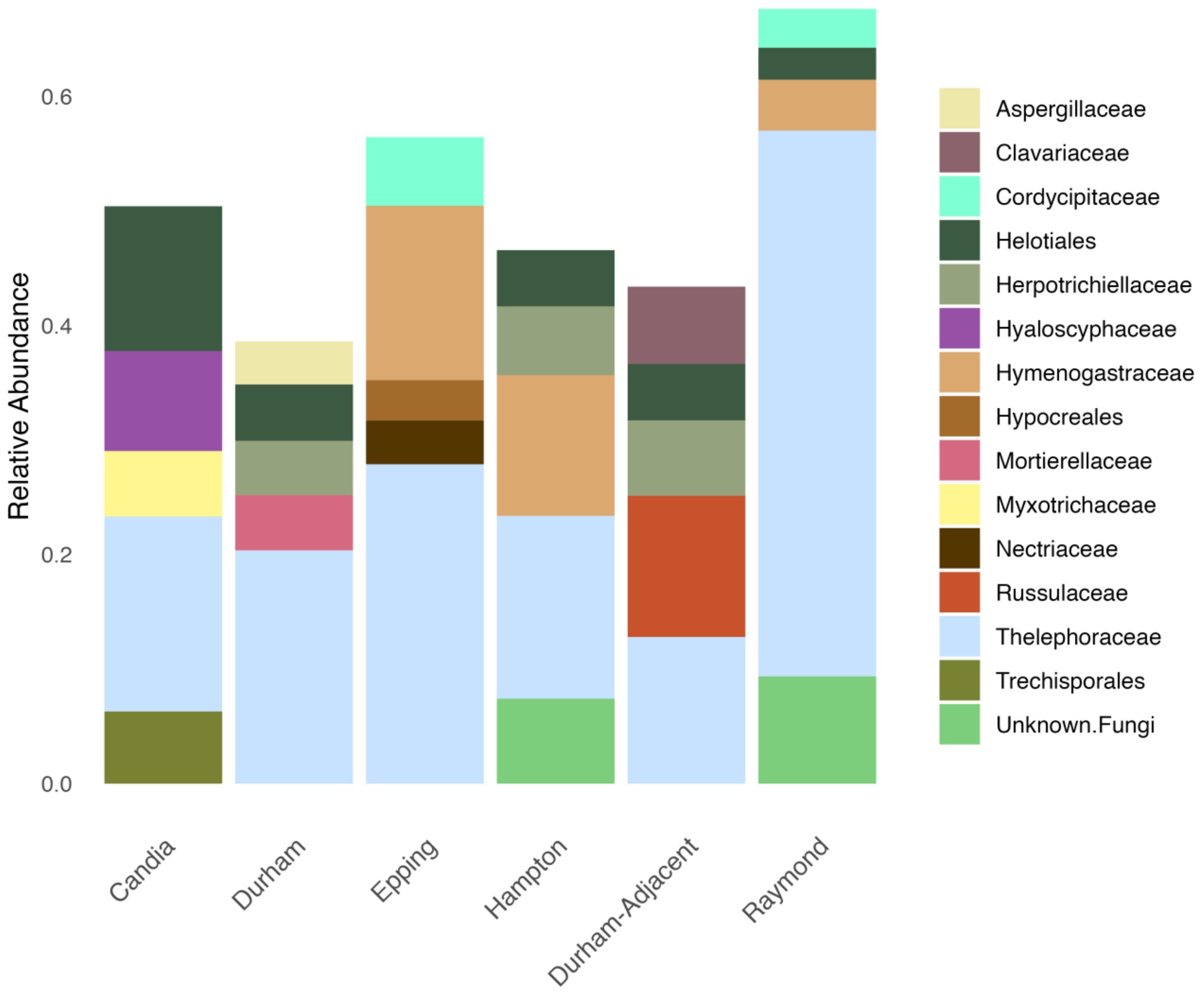

Figure S11. The top five fungal taxa with the highest average relative abundance in the six sampling sites' alder rhizospheres. Only those nodule occupants that were consistently found in 100% of rhizosphere samples for each site were considered, then the top five fungal taxa were plotted for simplicity.

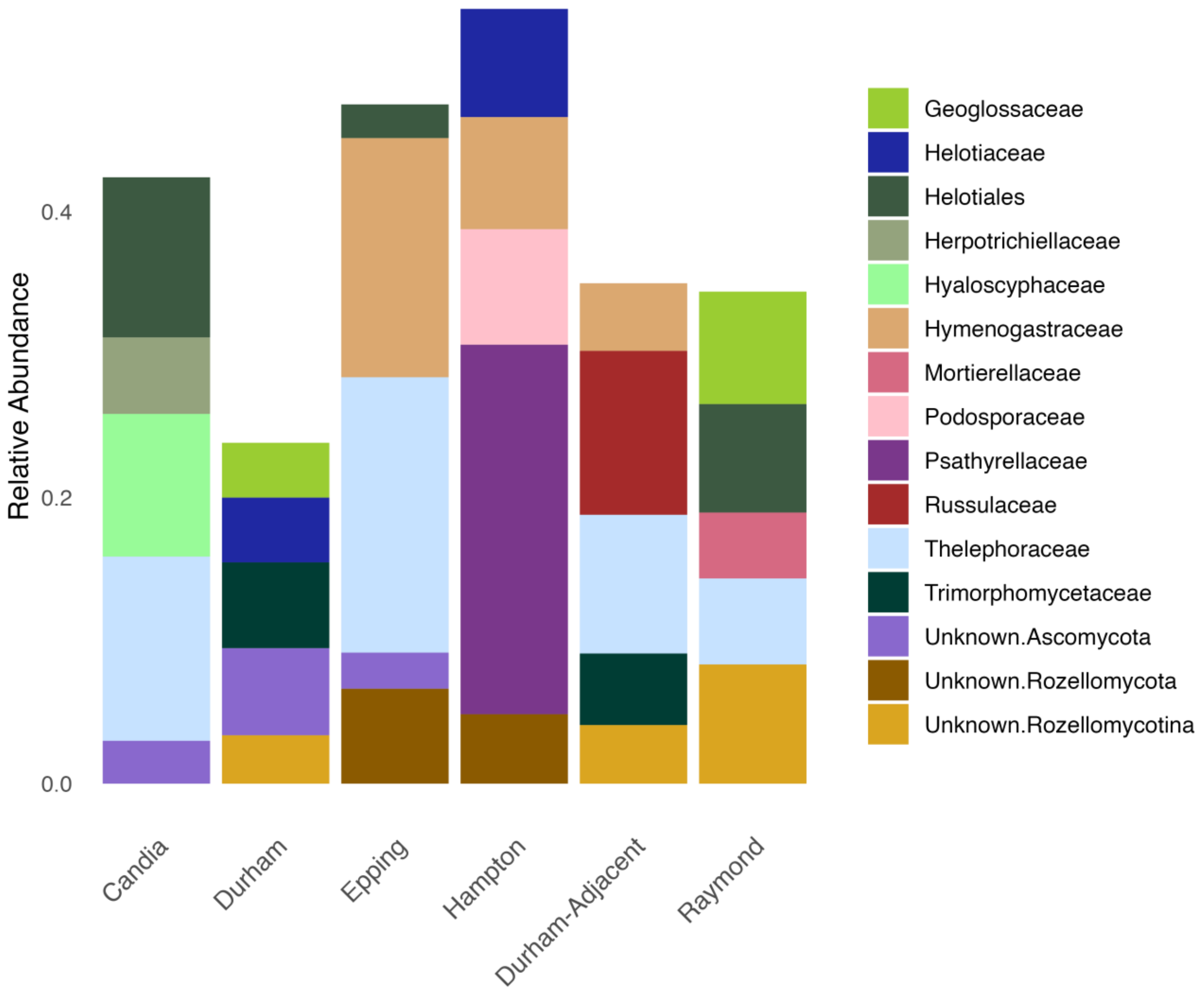

Figure S12. The top five fungal taxa with the highest average relative abundance in the six sampling sites' bulk soils. Only those soil occupants that were consistently found in 100% of soil samples for each site were considered, then the top five fungal taxa were plotted for simplicity.

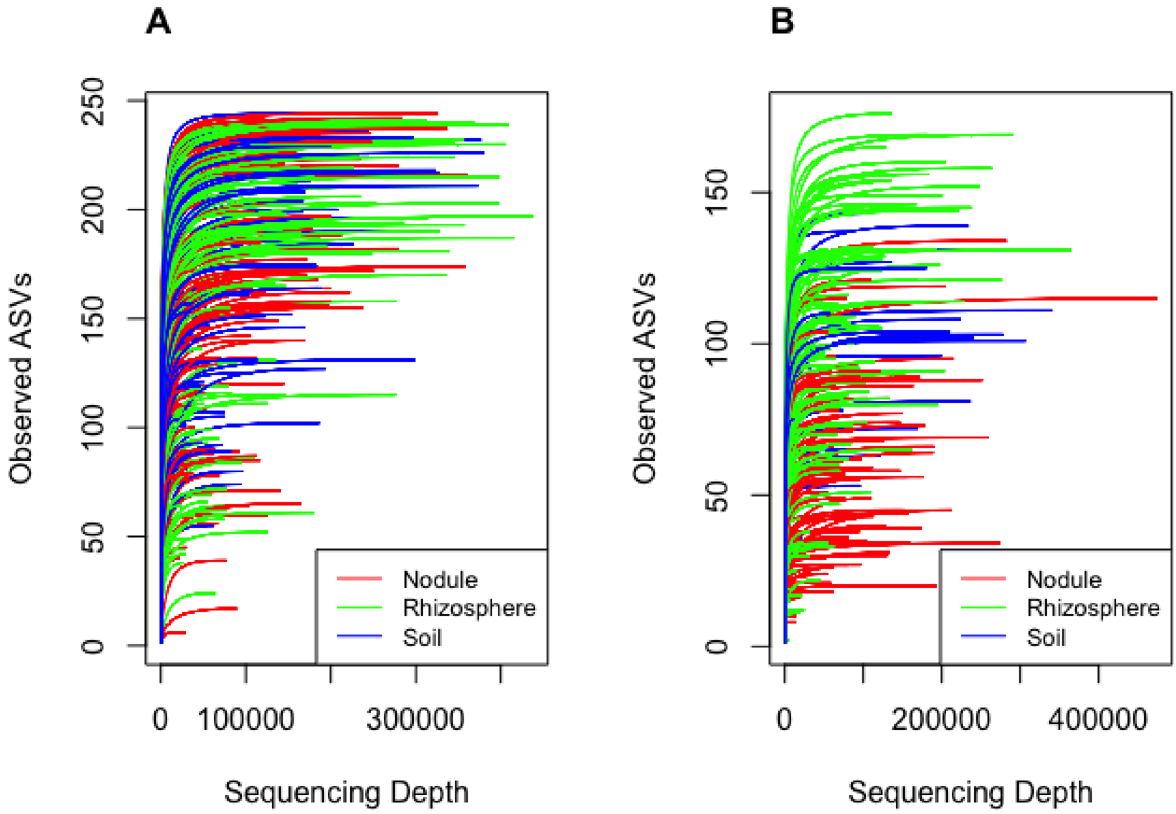

Figure S13. Alpha rarefaction curves of observed ASVs across sequencing depths for the filtered bacterial (A) and fungal (B) communities. The communities were filtered by removing samples with less than 20,000 reads and 4000 reads for the bacterial and fungal communities, respectively. Colors indicate sample type. Rarefaction to 20,000 reads and 4000 reads for the bacterial and fungal communities, respectively, was done for downstream alpha diversity analyses.
